# Polymeric Micelles Based on Modified Glycol Chitosan for Paclitaxel Delivery: Preparation, Characterization and Evaluation

**DOI:** 10.3390/ijms19061550

**Published:** 2018-05-23

**Authors:** Na Liang, Shaoping Sun, Xianfeng Gong, Qiang Li, Pengfei Yan, Fude Cui

**Affiliations:** 1 Key Laboratory of Photochemical Biomaterials and Energy Storage Materials, Heilongjiang Province, College of Chemistry & Chemical Engineering, Harbin Normal University, Harbin 150025, China; liangna528@163.com; 2 Key Laboratory of Chemical Engineering Process & Technology for High-Efficiency Conversion, College of Heilongjiang Province; School of Chemistry and Material Science, Heilongjiang University, Harbin 150080, China; gongxianfeng@sina.com (X.G.); liqianghlj100@163.com (Q.L.); 3 Key Laboratory of Functional Inorganic Material Chemistry, Heilongjiang University, Harbin 150080, China; 4 School of Pharmacy, Shenyang Pharmaceutical University, Shenyang 110016, China; syphucuifude@163.com

**Keywords:** glycol chitosan, α-tocopherol succinate, amphiphilic polymer, micelles, paclitaxel

## Abstract

Amphiphilic polymer of α-tocopherol succinate modified glycol chitosan (TS-GC) was successfully constructed by conjugating α-tocopherol succinate to the skeleton of glycol chitosan and characterized by Fourier-transform infrared (FT-IR) and proton nuclear magnetic resonance (^1^H-NMR). In aqueous milieu, the conjugates self-assembled to micelles with the critical aggregation concentration of 7.2 × 10^−3^ mg/mL. Transmission electron microscope (TEM) observation and dynamic light scattering (DLS) measurements were carried out to determine the physicochemical properties of the micelles. The results revealed that paclitaxel (PTX)-loaded TS-GC micelles were spherical in shape. Moreover, the PTX-loaded micelles showed increased particle sizes (35 nm vs. 142 nm) and a little reduced zeta potential (+19 mV vs. +16 mV) compared with blank micelles. The X-ray diffraction (XRD) spectra demonstrated that PTX existed inside the micelles in amorphous or molecular state. In vitro and in vivo tests showed that the PTX-loaded TS-GC micelles had advantages over the Cremophor EL-based formulation in terms of low toxicity level and increased dose, which suggested the potential of the polymer as carriers for PTX to improve their delivery properties.

## 1. Introduction

Paclitaxel (PTX), as a powerful anti-tumor drug, has been extensively used in the clinical treatment of several solid tumors, such as refractory ovarian cancer, metastasis breast cancer, non-small cell lung cancer, Acquired Immune Deficiency Syndrome-related Kaposi’s sarcoma and other cancers [1,2]. Due to its poor water solubility of approximately <2 μg/mL, PTX is currently solubilized in a 50:50 mixture of Cremophor EL (PEG-35 caster oil) and dehydrated ethanol as Taxol^®^ (Bristol-Myers Squibb, New York City, NY, USA). However, several studies reported that Cremophor EL induced serious side effects such as hypersensitivity, nephrotoxicity, neurotoxicity, and the extraction of plasticizer from the infusion tubes [3]. In light of these drawbacks, a number of alternative preparations were investigated, including liposomes, nanocrystals, micelles, cyclodextrin complexes and PTX conjugates [4,5,6,7,8].

Among these formulations, polymeric micelles have been proven as promising drug delivery systems for PTX administration [9,10], because of their attractive characteristics, such as biocompatibility, high drug-loading content, small size (<200 nm) and propensity to evade scavenging by the mononuclear phagocyte system (MPS) [11,12]. Moreover, the nanoscale dimensions of polymeric micelles permit their selectively accumulation in tumor tissues due to the enhanced permeability and retention (EPR) effect, which is termed “passive targeting” [13,14]. The formation of polymeric micelles is generally considered as the self-assembly of polymeric amphiphiles. In aqueous medium, polymeric amphiphiles form the micelles consisting of the inner hydrophobic core and the outer hydrophilic shell. The hydrophobic core provides a storeroom for loading hydrophobic drugs, and the hydrophilic shell allows retaining the stability of micelles in an aqueous environment [15].

Due to its favorable properties, such as biodegradability, biocompatibility, nontoxicity and bioadhesivity, chitosan has been widely studied as a pharmaceutical carrier for drug delivery [16,17]. However, chitosan is insoluble at pH values above its pKa (6.4) in water, and this obviously limits its biomedical applications.

In recent years, glycol chitosan (GC), which possesses good solubility over a broad range of pH, has been studied to construct drug delivery systems for PTX, such as nanocrystals, nanoparticles, hydrogels and microspheres [18,19,20,21]. The hydrophobically modified GC, which could be used as micellar carriers, has been extensively studied [22,23].

α-Tocopherol is a good solvent for many hydrophobic drugs because of its excellent lipophilic nature [24]. Once it is grafted on to the backbone of GC, it may serve as the hydrophobic segment and therefore provide sufficient capacity for poorly soluble drugs.

Inspired by the above investigations, in this study, an amphiphilic polymer α-tocopherol succinate modified glycol chitosan (TS-GC) was designed for PTX delivery. The TS-GC was prepared through amide formation. The preparation, characterization and self-assembling ability of TS-GC were studied. Furthermore, the physicochemical properties, hemolysis, in vitro cytotoxicity and in vivo antitumor activity of PTX-loaded micelles were evaluated deeply.

## 2. Results and Discussion

### 2.1. Synthesis and Characterization of TS-GC

**Figure 1:**
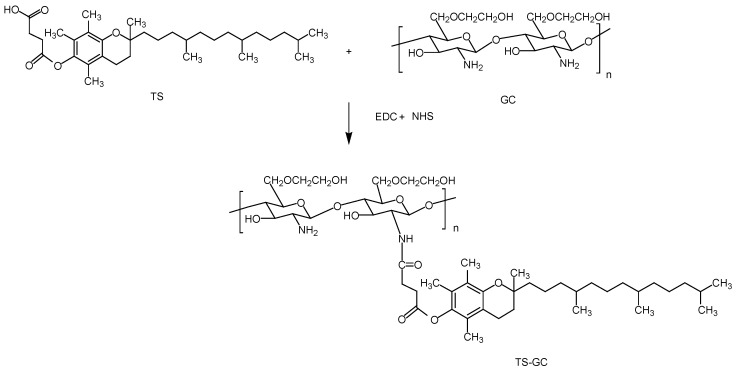
Synthesis of α-tocopherol succinate modified glycol chitosan (TS-GC).

In this study, the polymer TS-GC was synthesized via the coupling reaction between carboxyl group of TS and amine group of GC in the presence of water-soluble 1-Ethyl-3-(3-dimethylaminopropyl) carbodiimide hydrochloride (EDC). Firstly, an active ester intermediate was formed between the carboxyl group of TS and EDC. Then the amino bond can easily formed by reaction of primary amino of GC with the intermediate. NHS was used to stabilize the intermediate to achieve higher yield [25]. The scheme of the reaction between GC and TS was shown in Figure 1.

**Figure 2:**
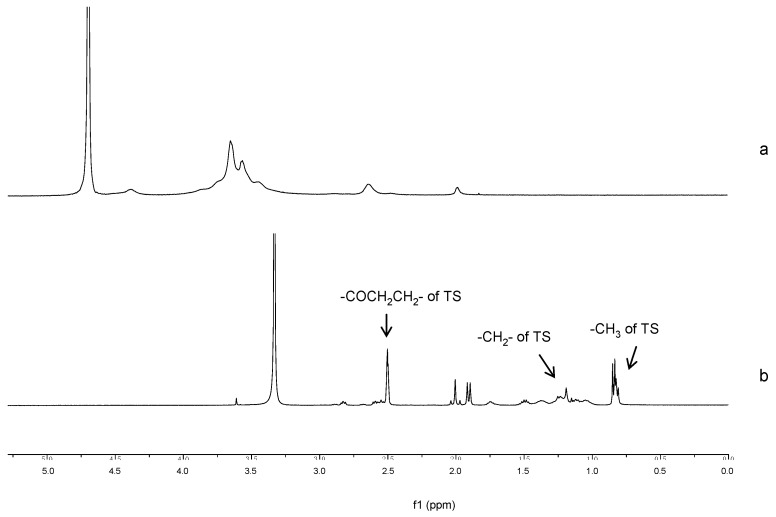
Proton nuclear magnetic resonance (^1^H-NMR) spectra of (**a**) glycol chitosan (GC) and (**b**) TS-GC.

^1^H-NMR spectra confirmed the grafting of TS chain onto GC as illustrated in Figure 2. In the spectrum of TS-GC, the proton peaks of TS, including methyl (0.77–0.88 ppm) and methylene (0.99–1.54 ppm) that belonged to the protons of the long-chain alkyl group of TS were observed. Moreover, the new-emerged signals at 2.45–2.63 ppm were attributed to the methene hydrogen (–COCH_2_CH_2_–) of the succinyl group of TS [26]. These spectra proved the formation of TS-GC.

**Figure 3:**
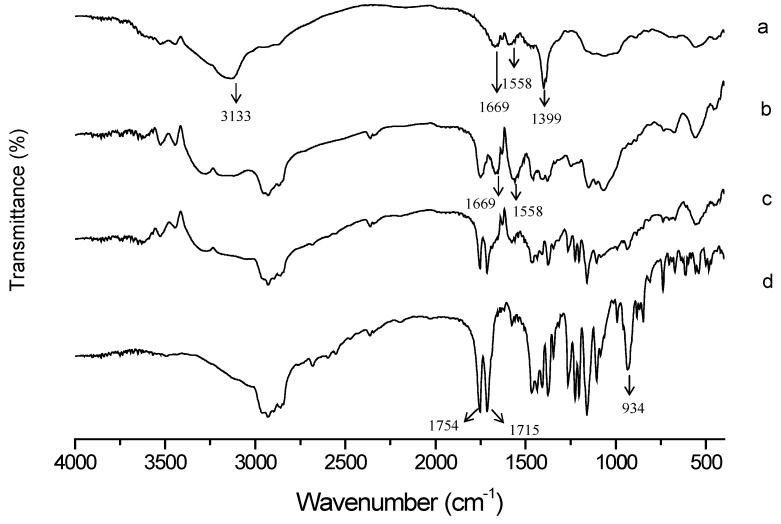
Fourier-transform infrared (FT-IR) spectra of (**a**) GC, (**b**) TS-GC, (**c**) physical mixture of GC and TS, and (**d**) TS.

FT-IR was used to characterize the functional groups of GC before and after modification. The FT-IR spectra of GC (a), TS-GC (b), physical mixture of GC and TS (c) and TS (d) are shown in Figure 3. In curve a, GC showed characteristic signals at 3133 cm^−1^ (O–H stretch overlapped with N–H stretch), 1669 cm^−1^ (amide I band, C=O stretch of acetyl group), 1558 cm^−1^ (amide II band, N–H bending) and 1399 cm^−1^ (C–H bending), respectively. The spectrum of TS (curve d) showed characteristic peaks of C=O bond at 1754 cm^−1^ (the carbonyl of ester bond) and at 1715 cm^−1^ (carboxylic C=O). And the peak at 934 cm^−1^ was attributed to the out-of-plane bending vibrations of carboxylic C–OH. Compared with GC, the increased intensity of peaks at 1669 cm^−1^ and 1558 cm^−1^ in TS-GC (curve b) indicated the formation of amide bond. In addition, disappearance of carboxylic C=O signal at 1715 cm^−1^ and carboxylic C–OH signal at 934 cm^−1^ further confirmed full reaction of TS with GC. All the above indicated the successful introduction of TS.

The degree of amino substitution was calculated by measuring the amount of terminal amino groups of TS-GC with 2,4,6-trinitrobenzene sulphonic acid (TNBS reagent). The maximum absorbance of the yellow colour product was at 344 nm, and the absorbance was in proportion to the number of primary amino groups. From the calibration curve obtained with unmodified GC solution with different concentrations, the substitution degree of TS-GC in this experiment was calculated as 11.3%.

Critical aggregation concentration (CAC) plays an important role in maintaining the stability of micelles upon dilution. Only when the concentration of the polymer is higher than its CAC can micelles be formed. Polymeric micelles are generally more stable because of their markedly lower CAC. For the determination of the CAC of TS-GC, the pyrene fluorescence was employed to monitor the properties of TS-GC in solution. This method utilized pyrene’s sensitivity to the local polarity of the environment. Below the CAC, the polymers only exist as single chains, and pyrene is solubilized in water. When the concentration increases to reach the critical value called CAC, polymer chains start to associate to form micelles, and pyrene partitions preferentially toward the hydrophobic cores of micelles. This leads to the increase of fluorescence intensity, and the intensity of the third peak increased significantly compared to that of the first peak. So the CAC values were calculated from curves of I_3_/I_1_ versus polymer concentration. The CAC was defined as the intercept of the tangents to the curve before and after the point of inflection [27]. For TS-GC, the CAC was calculated to be 7.2 × 10^−3^ mg/mL, which was significantly lower than that of the low molecular weight surfactants in water. This implied the TS-GC micelles could be stable and not easily dissociate upon dilution.

### 2.2. Preparation of TS-GC Micelles

In general, the aggregates of amphiphilic polymers can be prepared through diafiltration or sonication methods. As for diafiltration, the polymer was dissolved in co-solvent and then dialyzed against water. For TS-GC, it was not easily dissolved in the mixture of organic solvent with water, so the probe-sonication was employed. Micelle formation is a delicate balance between the attractive force that leads to the association of molecules and the repulsive force that prevents unlimited growth of the micelles [28]. It was reported that once the micelle structure was formed completely, the drugs were hardly to be incorporated into the micelles [29]. So, in this study, in order to get high encapsulation efficiency, the PTX loading occurred simultaneously with self-assembly of TS-GC under the probe-sonication treatment as stated in Section 3.5. The PTX encapsulation efficiency reached to 71.8%, and the drug loading capacity was calculated as 8.0%.

### 2.3. Characterization of PTX-Loaded TS-GC Micelles

#### 2.3.1. XRD Analysis

**Figure 4:**
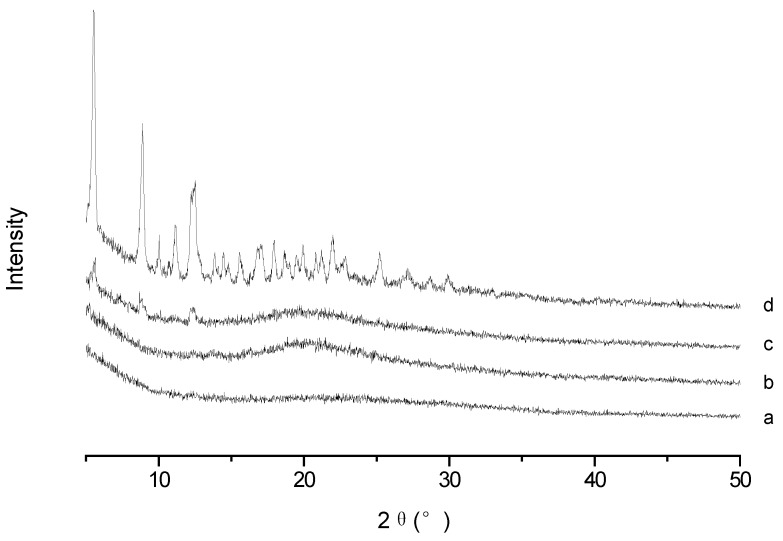
X-ray diffraction (XRD) spectra of (**a**) blank micelles; (**b**) paclitaxel (PTX)-loaded micelles; (**c**) physical mixture of PTX and blank micelles; and (**d**) PTX.

To confirm the existence form of PTX in PTX-loaded TS-GC micelles, XRD analysis was conducted for PTX, blank micelles, their physical mixture and PTX-loaded micelles. As illustrated in Figure 4, typical intense diffraction peaks of PTX were still observed with weak intensity in the pattern obtained from the physical mixture of PTX and blank micelles, which reflected the presence of PTX crystal in the mixture. While the spectrum of lyophilized PTX-loaded micelles was similar to that of the blank micelles, and there were no diffraction peaks for PTX. It can be concluded that PTX was encapsulated in the polymeric micelles in molecular or amorphous state and there was no free drug on the surface of micelles.

#### 2.3.2. Particle Size and Zeta Potential

**Figure 5:**
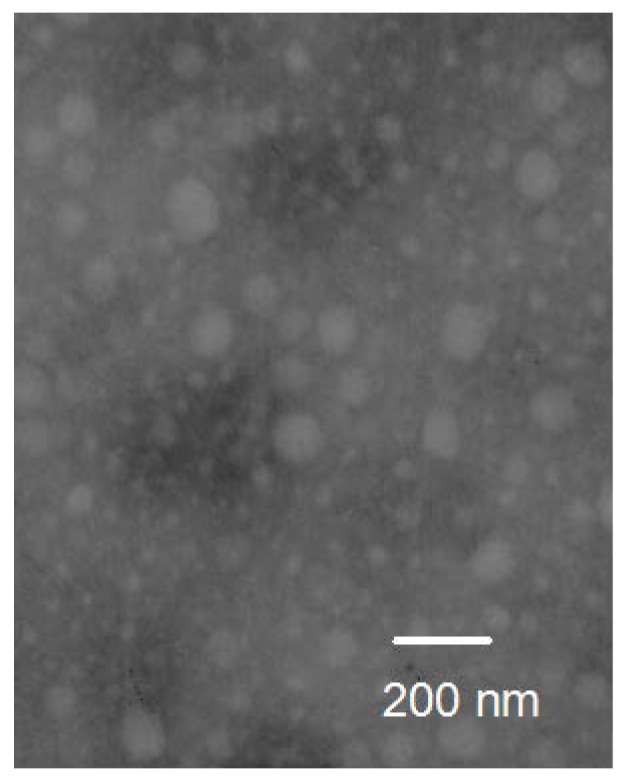
Transmission electron microscope (TEM) image of PTX-loaded micelles.

Size of the micelles is an important factor affecting the in vivo fate of the drug. In this study, the particle size and their distribution of the micelles were measured by dynamic light scattering method. From the result, the mean particle size of PTX-loaded micelles was 142 nm with polydispersity index (PDI) of 0.186, and it was larger than that of blank micelles (35 nm, PDI of 0.105), which indicated the encapsulation of PTX into the micelles. For both bare and PTX-loaded micelles, the size distribution was narrow. It was reasonably safe to assume an increasing accumulation of the drug in tumor tissue. Because it was reported that the small size of micelles (<200 nm) can reduce non-selective clearance by the reticuloendothelial system (RES) and show EPR effect for passive accumulation in certain tumor sites [30]. Moreover, the size-sieving may occur during the distribution process in the body, and the narrow size distribution may promote the selective accumulation at the target site. Transmission electron microscope (TEM) micrograph of PTX-loaded micelles is shown in Figure 5. It was obvious that the micelles were spherical in shape.

For a micellar solution, zeta potential can greatly influence the particle stability through the electrostatic repulsion. Relatively high surface charge could provide a repelling force between the particles, and thus increase the stability of the system. In this study, the blank micelles and PTX-loaded micelles in aqueous medium were positively charged with zeta potential of +19 mV and +16 mV, respectively, due to the presence of ionized amino groups of TS-GC distributing on the surface of micelles. It is reasonable to confirm the aqueous stability of the micelles.

### 2.4. In Vitro Hemolytic Test

As analogs of low molecular weight surfactants, amphiphilic polymers may solubilize lipids or be inserted into phospholipid membranes, and consequently lead to hemolysis of red blood cells following intravenous administration [31]. It is necessary to determine whether TS-GC induces hemolysis and is safe for intravenous injection. The hemolysis of PTX-loaded TS-GC micelles was compared with that of Cremophor EL-based formulation. It was observed that in the range of 10–200 μg/mL, the hemolysis of PTX-loaded TS-GC micelles was almost negligible, with only 4.2% at the concentration of 200 μg/mL, while the hemolysis induced by the Cremophor EL-based formulation increased from 0.04% to 10.9%. The results suggested that PTX-loaded micelles were not toxic to the erythrocytes.

### 2.5. In Vitro Cytotoxicity Study

**Figure 6:**
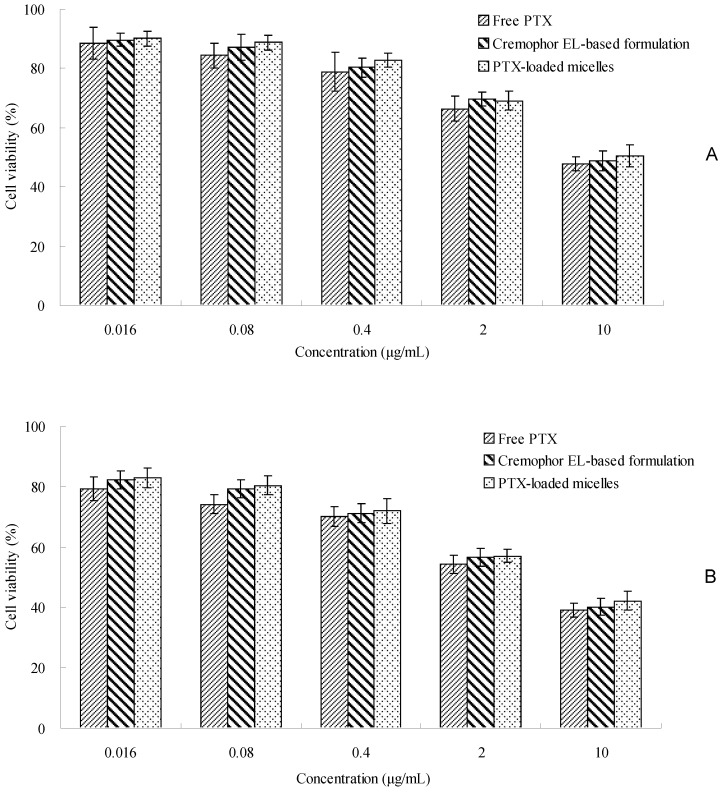
In vitro cytotoxicity of free PTX in dimethyl sulfoxide (DMSO), Cremophor EL-based formulation and PTX-loaded micelles against MCF-7 cells after 24 h (**A**) and 48 h (**B**) incubation (mean ± SD, *n* = 3).

The cytotoxicity study was conducted by 3-(4,5-dimethylthiazol-2-yl)-2,5-diphenyl tetrazolium bromide (MTT) method to assess the effectiveness of PTX-loaded TS-GC micelles. As shown in Figure 6, the cytotoxicity of PTX-loaded TS-GC micelles was similar to that of Cremophor EL-based formulation with equivalent doses in the concentration ranges used in this study. Furthermore, as the concentration and incubation time increased, PTX-loaded micelles and Cremophor EL-based formulation displayed increasing cytotoxicity. It was straightforward to understand that both concentration and incubation time played a major role in the in vitro cytotoxicity of PTX. For longer incubation periods, a larger number of cells enter the G2 and M cell cycle phases, during which PTX is more active. The findings suggested that PTX-loaded TS-GC micelles could be used as a potential PTX carrier.

### 2.6. In Vivo Antitumor Activity Study

**Figure 7:**
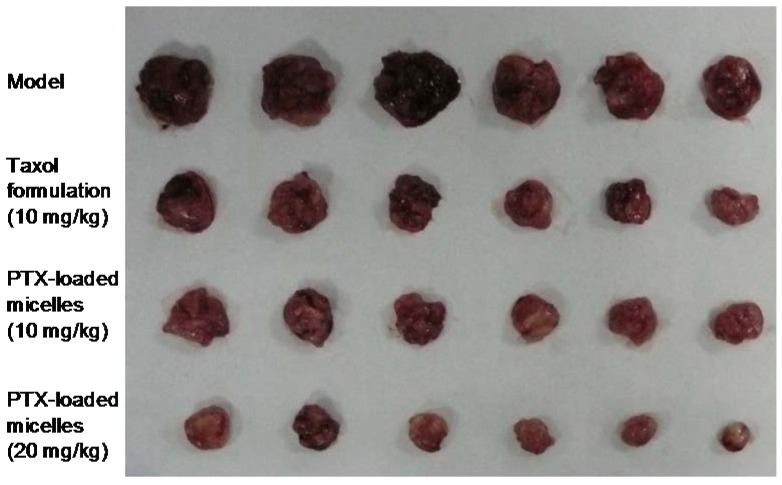
Photograph of tumors from each treatment group excised after intravenous injection treatment on day 13.

The antitumor efficacy of PTX-loaded micelles in vivo was consistent well with the in vitro cell experiment. As shown in Figure 7, comparing with the saline control, a striking antitumor response was observed in all the treatment groups (*p* < 0.05). Furthermore, PTX-loaded micelles and Cremophor EL-based formulation had the similar antitumor efficacy at the same dose of 10 mg/kg, with tumor inhibition rate (TIR) of 72.3% and 69.6%, respectively, and the difference between the two groups was not statistically significant. The slight superior of the micelles might be explained by the increased concentration of PTX in tumor tissue due to the EPR effect, and the exact mechanism will be further studied.

On the other hand, the toxic effect of PTX-loaded micelles was much less than that of the Cremophor EL-based formulation. At the high dose of 20 mg/mL, the intravenous administration of Cremophor EL-based formulation resulted in development of immediate ataxia and enhanced respiration, and 3 in 6 animals died although the injection speed was slowed down and the first aid treatment was given immediately after the symptoms appeared. In contrast, none of the mice treated with PTX-loaded micelles died throughout the study. This may be explained by the serious side effect of Cremophor EL. While for the micelles, all the components constructed TS-GC were nontoxic and biocompatible. It was obvious that the low toxicity of PTX-loaded micelles is of great value.

Taken together, these results implied that PTX-loaded TS-GC micelles not only had similar to better antitumor efficacy as Cremophor EL-based formulation, but also induced less systemic toxicity, especially at the higher dose. The PTX-loaded micelles might allow the administration of a higher dose of PTX.

## 3. Materials and Methods

### 3.1. Materials

Glycol chitosan (Mw 250 kDa, deacetylation degree of 82.7%), 2,4,6-trinitrobenzene sulfonic acid (TNBS), pyrene (purity > 99%) and 3-(4,5-dimethylthiazol-2-yl)-2,5-diphenyl tetrazolium bromide (MTT) were purchased from Sigma, St. Louis, MO, USA. 1-Ethyl-3-(3-dimethylaminopropyl) carbodiimide hydrochloride (EDC) and *N*-hydroxysuccinimide (NHS) were obtained from Shanghai Medpep Co., Ltd., Shanghai, China. α-Tocopherol succinate (TS) was kindly donated by Xinchang Pharmaceutical Co., Ltd., Shaoxing, China. Paclitaxel (PTX, purity of 99.9%) was supplied by Tianfeng Bioengineering Technology Co., Ltd., Shenyang, China. Cremophor EL was a kind gift from BASF Corp., Ludwigshafen, Germany. Dulbecco’s modified Eagle’s medium (DMEM), fetal bovine serum (FBS), and penicillin–streptomycin mixture were purchased from Gibco BRL, Carlsbad, CA, USA. All other chemicals and solvents were of analytical or chromatographic grade and used without further purification. Distilled water or Milli-Q water was used in all experiments.

### 3.2. Animals and Cell Line

The male New Zealand rabbit (weighing 2 kg) and specific pathogen-free female Kunming mice (5–6 weeks old, weighing 20–25 g) were acquired from Laboratory Animal Center of Harbin Medical University, Harbin, China. MCF-7 cells (human breast cancer cells) were obtained from American Type Culture Collection, Manassas, VA, USA. U14 cells (mouse uterine cervix carcinoma cells) were provided by the Cell Resource Center of Chinese Academy of Medical Sciences, Beijing, China. All animal procedures were performed in compliance with the animal care protocols approved by the Animal Ethics Committee of Harbin Medical University (18 May 2017, No. 201705180118).

### 3.3. Synthesis of α-Tocopherol Succinate Modified Glycol Chitosan (TS-GC)

α-Tocopherol succinate modified glycol chitosan (TS-GC) was synthesized by the coupling reaction between carboxyl group of TS with amine group of GC in the presence of EDC [32]. Briefly, GC (100 mg) was dissolved in 10 mL of distilled water and TS (64 mg) was dissolved in 10 mL of methanol. The TS solution was slowly added to the GC solution and followed by the addition of excessive EDC and NHS under gentle stirring at room temperature. After overnight reaction, the mixture was dialyzed against distilled water using a cellulose membrane (Viskase, Willowbrook, IL, USA, MWCO: 7000), and TS-GC was obtained by freeze-drying of the dialyzed solution.

### 3.4. Characterization of TS-GC

To confirm the formation of TS-GC, proton nuclear magnetic resonance (^1^H-NMR) analysis was operated at 300 MHz using Bruker Avance spectrometer (AV-300, Bruker, Karlsruhe, Germany). GC and the conjugate were dissolved in D_2_O and DMSO-d6 at the concentration of 1% (*w*/*v*), respectively.

In order to further investigate the structural changes during the synthesis process, Fourier-transform infrared (FT-IR) spectra of GC, TS, their physical mixture and the conjugate were obtained on FT-IR spectrometer (Tensor II, Bruker, Fällanden, Switzerland) in the range between 4000 and 400 cm^−1^ after compressed into KBr pellets.

The degree of substitution means the number of tocopherol groups per 100 anhydroglucose units (amino groups) of TS-GC. The amount of remaining terminal amino residues on TS-GC was measured using 2,4,6-trinitrobenzene sulfonic acid (TNBS reagent) according to TNBS method [33,34]. The numerical value was calculated from the calibration curve that obtained by GC solution.

The critical aggregation concentration (CAC) of TS-GC in aqueous milieu was measured using a fluorometer (F-2500 FL Spectrophotometer, Hitachi Ltd., Tokyo, Japan). The pyrene fluorescence was monitored to evaluate the micropolarity and hydrophobicity of the region in which it was solubilized, so as to prove the formation of the micelles [35].

### 3.5. Preparation of PTX-Loaded TS-GC Micelles

PTX-loaded TS-GC micelles were prepared by a probe-type ultrasonic method. Briefly, 10 mg of TS-GC was swollen in 10 mL of distilled water under gentle stirring overnight at room temperature to ensure complete dispersion. PTX solution was prepared by dissolving PTX in methanol at the concentration of 1 mg/mL. The PTX solution was added into the TS-GC solution, and the final mixture was sonicated for 10 min at 400 W in an ice bath by a ultrasonicator (JY92-II, Ningbo Scientz Biotechnology Co., Ltd., Ningbo, China). The sonication was carried out with the pulse function (turned on for 3 s and off for 2 s). The unloaded PTX was removed by centrifugation at 4000 rpm for 10 min. The resulting supernatant was lyophilized to obtain PTX-loaded TS-GC micelles. The blank micelles were prepared by the same procedure except no PTX was added.

### 3.6. Characterization of PTX-Loaded TS-GC Micelles

#### 3.6.1. X-ray Diffraction (XRD) Analysis

X-ray diffraction diagrams were detected using an X-ray diffractometer (Geigerflex, Rigaku Co., Akishima, Japan) with Cu Kα radiation. Samples were scanned from 5 to 50° (2θ) at a scanning speed of 2°/min and step size of 0.02°. The X-ray system was operated at a potential of 30 kV and current of 30 mA.

#### 3.6.2. Transmission Electron Microscopy (TEM) Observation

Morphology of the micelles was observed using a transmission electron microscope (TEM) (Jeol JEM1200EX, Tokyo, Japan) that operated at an accelerating voltage of 60 kV. For TEM, an aqueous droplet of micelles was immobilized on copper grids and negatively stained with phosphotungstate solution (2%, *w*/*v*), then dried at room temperature before observation.

#### 3.6.3. Measurement of Particle Size and Zeta Potential

The number-weighted diameter, their distribution, and zeta potential of the micelles were measured using photon correlation spectroscopy with a Zetasizer Nano-ZS90 at 25 °C. The lyophilized samples were suspended in distilled water before measurement.

#### 3.6.4. Determination of Drug Loading and Drug Encapsulation Efficiency

The drug loading and entrapment efficiency of the PTX-loaded micelles were determined as follows: 100 μL of PTX-loaded TS-GC micelles solution (concentration of TS-GC = 1.0 mg/mL) was centrifuged at 10,000 rpm for 10 min with ultrafilter (Vivaspin 500, MWCO 10 k, Sartorius Co., Göttingen, Germany). The unentrapped PTX amount in the ultrafiltrate (*W*_1_) was determined using HPLC method. The HPLC system consisted of a mobile phase delivery pump (LC-10ATVP HPLC pump, Shimadzu, Kyoto, Japan) and a UV detector (SPD-10A UV/Vis detector, Shimadzu, Japan). For separation, a Diamonsil^TM^ C_18_ reverse-phase column (200 × 4.6 mm, 5 μm, Dikma technologies Inc., Beijing, China) was used. The mobile phase consisted of a mixture of acetonitrile and water (60:40, *v*/*v*). The detector wavelength, column temperature, and flow rate of the mobile phase were set at 227 nm, 30 °C and 1.0 mL/min, respectively. The injection volume of the test samples was 20 µL.

In order to measure the total PTX content (*W*_0_) in the micelles, another 100 μL of identical PTX-loaded micelles solution was ultrasonicated in 10 mL methanol to extract PTX from the micelles. The encapsulation efficiency (EE%) and drug loading (DL%) of PTX were calculated using the following Equations (1) and (2):

EE% = (*W*_0_ − *W*_1_)/*W*_0_ × 100% (1)

DL% = (*W*_0_ − *W*_1_)/(*W*_0_ − *W*_1_ + 100) × 100% (2)

where *W*_1_ is the PTX content in the ultrafiltrate, and *W*_0_ is the total PTX amount in the solution. The unit was microgram.

### 3.7. In Vitro Hemolysis Test

Hemolysis assessment of the TS-GC micelles was conducted as the method reported by Gong et al. [36]. The rabbit blood, freshly drawn from the ear vein, was centrifuged at 3000 rpm for 10 min to isolate the erythrocytes. The erythrocytes were washed with normal saline for three times to make the supernatant achromatic, and then suspended in normal saline to get a 2% (*v*/*v*) suspension. The lyophilized powder of PTX-loaded TS-GC micelles was dispersed in 0.9% NaCl, and different amounts of micelle solution were added into the tubes with 2.5 mL of 2% erythrocyte dispersion in each. Then adequate amounts of normal saline were added in every tube to obtain a final volume of 5 mL. After incubating at 37 °C for 4 h, the mixture was centrifuged at 3000 rpm for 10 min to remove intact red blood cells (RBC). The supernatant was analyzed for released hemoglobin at 540 nm using a spectrophotometer (UV-Vis Spectrophotometer Model 752, Shanghai Spectrum Instruments Co., Ltd., Shanghai, China). To obtain 0 and 100% hemolysis, 2.5 mL of saline and 2.5 mL of distilled water was added to 2.5 mL of RBC suspension, respectively. The degree of hemolysis was calculated by the following Equation (3).

Hemolysis (%) = (*A_sample_* − *A*_0%_)/(*A*_100%_ − *A*_0%_) × 100% (3)

where *A_sample_*, *A*_0%_, and *A*_100%_ are the absorbance of the samples, a solution of 0% hemolysis, and a solution of 100% hemolysis, respectively.

### 3.8. In Vitro Cytotoxicity

The in vitro cytotoxic activity of samples was evaluated by MTT method using MCF-7 cell line [37]. Briefly, 50 μL of MCF-7 cells growing in the logarithmic phase were cultured in Dulbecco’s modified Eagle’s medium (DMEM) containing 10% (*v*/*v*) fetal bovine serum, 100 IU/mL of penicillin G sodium and 100 μg/mL of streptomycin sulfate. The cells were seeded in a 96-well microtitre plate at the density of 1 × 10^4^ cells per well and maintained in an incubator supplied with 5% CO_2_ at 37 °C. After reaching 75% confluence, the cells were incubated with free drug in dimethyl sulfoxide (DMSO), Cremophor EL-based PTX formulation and PTX-loaded TS-GC micelles at the equivalent drug concentrations ranging from 0.016 to 10 μg/mL for 24 and 48 h. At designated time intervals, the medium was removed and the wells were washed with PBS for two times. Then, 10 μL of MTT (5 mg/mL in PBS) was added to the wells. After an additional 4 h of incubation, the MTT medium was aspirated off and 100 μL of DMSO was added to each well to dissolve the formazan crystals. The absorbance was measured at 570 nm with a microplate reader (Bio-Tek Instruments Inc., Vernusky, VT, USA). Untreated cells were taken as the control with 100% viability, and cells without addition of MTT were used as blank to calibrate the spectrophotometer to zero absorbance. The cytotoxicity was calculated as follows Equation (4):

Cytotoxicity = (A_culture medium_ − A_sample_)/A_culture medium_ × 100% (4)

where A_culture medium_ and A_sample_ are the absorbance of cells incubated with culture medium and absorbance of cells exposed to the sample, respectively.

### 3.9. In Vivo Antitumor Activity

Specific pathogen-free Kunming mice, 5 to 6 weeks old, weighing 20–25 g were used for this study. The animals were housed six per cage in standard size cages under standard laboratory conditions (21 ± 2 °C, 12-h light: 12-h dark cycle, relative humidity of 50–60%) and allowed to access sterilized food and water freely. The mice were left to acclimatize for a week prior to the experiment.

U14 cells of the third passage in vivo were used for tumor development. Animals were inoculated with 2.0 × 10^6^ cells (0.2 mL/mouse) subcutaneously in the armpit of right anterior limb. Three days later, the tumors were palpable, and the tumor model was established. The animals were randomized and divided into different groups (*n* = 6): (1) normal saline group (negative control); (2) Cremophor EL-based PTX formulation groups (positive control, 10 mg/kg and 20 mg/kg); and (3) PTX-loaded TS-GC micelles groups (10 mg/kg and 20 mg/kg), and the treatments were initiated. All samples were injected intravenously via the tail vein every 3 days, four times in total. The day that mice received treatment was set as day 1. At the end of the experiment, on day 13, the animals were sacrificed by cervical dislocation, and the tumors were excised, weighed and imaged. The tumor inhibition rate (TIR) of each formulation was defined as follows Equation (5):

TIR = (tumor weight of negative control group − tumor weight of treatment group)/tumor weight of negative control group × 100%(5)

### 3.10. Statistical Analysis

Each experiment was performed in triplicate. Values were expressed as mean±standard deviation (SD). Statistical data analysis was performed using the Student’s *t*-test with *p* < 0.05 as the level of significance.

## 4. Conclusions

In this study, a novel amphiphilic derivative of glycol chitosan was successfully synthesized by grafting α-tocopherol succinate onto the skeleton of glycol chitosan. In aqueous milieu, the conjugates provide stable self-aggregates above the CAC. Furthermore, the water-insoluble anticancer agent, PTX, was successfully encapsulated into the core of the micelles. The mean diameters of PTX-loaded micelles were about 142 nm, which was larger than blank ones. The spherical morphology of the micelles was visually confirmed by TEM. Moreover, the PTX-loaded TS-GC micelles possessed antitumor activities in vitro and in vivo, and had advantages over the commercially available Cremophor EL-based formulation in terms of low toxicity levels and increased tolerated dose. Therefore, this novel TS-GC polymer might be used as a potential carrier for PTX.
